# Parathyroid Hormone Fluctuations During Thyroid and Parathyroid Surgery

**DOI:** 10.1002/oto2.70068

**Published:** 2025-01-17

**Authors:** Emily S. Sagalow, Yuna Kim, Shirley Wong, Robert C. Wang

**Affiliations:** ^1^ Department of Otolaryngology–Head and Neck Surgery University of Nevada Las Vegas School of Medicine Las Vegas Nevada USA

**Keywords:** parathyroid hormone, parathyroidectomy, perioperative, stress hormone, thyroidectomy

## Abstract

**Objective:**

Stress hormone levels such as cortisol and epinephrine increase with general anesthesia (GA) and surgery. Parathyroid hormone (PTH) has been shown to increase with GA in those undergoing parathyroidectomy (PT) with abnormal parathyroid function, but there are conflicting reports of it in those with normal parathyroid function. In this study, we aim to determine the effects of anesthetic and surgical stress on those with abnormal parathyroid function undergoing PTs as well as those with normal parathyroid function undergoing unilateral/total thyroidectomies (UTs/TTs).

**Study Design:**

Prospective study.

**Setting:**

Single tertiary academic center.

**Methods:**

Patients undergoing TT, UT, and PT were studied. PTH was measured consecutively during the preoperative, postanesthetic induction before incision, intraoperative, and postoperative periods.

**Results:**

One hundred sixty patients were included, with 77 and 31 undergoing TT and UT, respectively, and 52 undergoing PT. Mean PTH levels were significantly higher following induction and intubation across all groups (TT: 139.2 vs 65.1 pg/mL, 113.8% increase; UT: 130.4 vs 57.1 pg/mL, 128.4% increase; PT: 219.6 vs 163.7 pg/mL, 34.1% increase) and remained elevated until excision (TT: 131.8 pg/mL; UT: 124.9 pg/mL; PT: 228.7 pg/mL). Following UT, mean PTH declined to preoperative levels by 1 hour postexcision. Compared to thyroidectomy groups, PTH in the PT group showed more variable responses to anesthesia induction.

**Conclusion:**

PTH consistently increases in response to anesthetic and surgical stress in adults undergoing UT and TT with normal preoperative parathyroid function. In contrast, those with hyperparathyroidism demonstrated variable changes.

Parathyroid hormone (PTH) is a hormone secreted by parathyroid glands that plays an essential role in calcium homeostasis. PTH has a short half‐life of approximately 5 minutes and therefore is an ideal real‐time indicator of parathyroid gland function.^1^ Catecholamines, such as epinephrine, are known to stimulate PTH secretion, resulting in changes in serum calcium concentrations.^2^, ^3^, ^4^ Administering general anesthesia (GA) during surgery has been shown to evoke a catecholamine response leading to an elevation in intraoperative parathyroid hormone (IOPTH).^2^ Monitoring IOPTH levels is important in thyroidectomy to monitor parathyroid gland function and predict postthyroidectomy hypocalcemia risk.^5^, ^6^, ^7^, ^8^, ^9^, ^10^ In addition to calcium regulation, PTH also has inotropic and vasodilatory effects as well as a relationship with the renin‐angiotensin‐aldosterone system.^11^ The multiple roles PTH has to demonstrate its significance in decision making during and after surgery.^12^, ^13^, ^14^, ^15^ Therefore, it is important to understand the different factors that can affect PTH levels intraoperatively.

There have been few studies investigating how preoperative parathyroid function can affect PTH levels intraoperatively. Cinamon et al found that GA and endotracheal intubation caused a significant PTH increase in patients with hyperparathyroidism and only a mild increase in healthy adults.^16^ However, they measured PTH levels in healthy adults undergoing various elective, nonhead, and neck surgeries with distinct anesthesia methods. The different nature and severity of the surgeries could result in varying levels of stress hormones and their effect on PTH. Mahajna et al reported that there were only mild elevations in PTH in patients with hyperparathyroidism after undergoing GA and endotracheal intubation.^17^ The conflicting results from these studies demonstrate the need to further investigate how PTH responds to anesthetic and surgical stress in patients with normal parathyroid function versus patients with abnormal parathyroid function.

In this study, we aim to determine the effects of anesthetic and surgical stress on those with abnormal parathyroid function undergoing parathyroidectomies (PTs) as well as those with normal parathyroid function undergoing unilateral/total thyroidectomies (UTs/TTs).

## Methods

### Study Design and Patient Selection

Consecutive patients who underwent TT, UT, or PT performed by a single surgeon at a tertiary academic center and a community hospital between December 2014 and February 2017 were included in this prospective cohort study. This study was approved by the University of Nevada, Las Vegas, Institutional Review Board. Consecutive PT for primary hyperparathyroidism and thyroidectomy cases were included during the study timeframe. One case of hyperparathyroidism with end‐stage renal failure was excluded. All patients were orotracheally intubated, without paralyzation due to electromyography monitoring performed in all cases, and given Sevoflurane, Sufentanil, and Propofol.

### PTH Measurements

The main outcome measure was PTH measurements at various timepoints pre‐, intra‐, and postoperatively to determine the effects of anesthetics and surgery on patients with normal parathyroid function undergoing unilateral thyroidectomy, TT, or PT. PTH was measured consecutively at the following time points: preoperative (in the preoperative holding area), preincision (following anesthesia induction and endotracheal intubation but prior to skin incision), pre‐excision (prior to thyroid or parathyroid gland excision), 5 and 10 minutes postexcision (PT group only), 20 minutes and 1 hour postexcision (TT or UT group only), and 6 and 12 hours postoperatively (TT group only). All intraoperative PTH measurements were performed using arterial blood samples. PTH was measured using 1 of 2 calibrated, automated central laboratory‐based intact PTH analyzers: (1) ARCHITECT i2000SR Intact PTH Assay (Abbott Diagnostics) and (2) ADVIA Centaur XP Immunoassay System (Siemens Healthcare Diagnostics). The margin of error for the PTH assay analyzer was ≤4%. Normal intact PTH was defined as 11.1 to 79.5 pg/mL.

### Other Outcome Measures

Additional variables collected included serum calcium, 25‐OH vitamin D, albumin, and estimated glomerular filtration rate (eGFR). Albumin‐corrected total calcium level of 7.6 mg/dL or less collected at 12 hours postoperatively was considered significant postoperative hypocalcemia.^18^ Clinical symptoms of hypocalcemia, such as perioral/extremity paresthesia and Chovstek's sign were noted. Postoperative calcium supplementation was only give if post‐excision PTH was less than 10 pg/mL or the patient exhibited symptoms of clinical hypocalcemia. Stages of chronic kidney disease (CKD) were determined by eGFR: Stages I (eGFR ≥ 90 mL/min/1.73 m^2^; normal), II (eGFR 60‐89), III (eGFR 30‐59), IV (eGFR 15‐29), and V (eGFR <15 or on dialysis).

### Statistical Analysis

Statistical analysis was performed using SPSS Version 24.0 (IBM Corp), GraphPad Prism Version 7 (GraphPad Software), and Microsoft Excel 2016 (Microsoft Office). The level of statistical significance was set at *P* = .05. Student's *t* test was utilized to compare PTH levels between thyroidectomy types (TT and UT) and among different time points. One‐way analysis of variance testing was utilized to determine significance among all 3 surgery groups (reported as *F* ratio). For assessing the correlation among PTH levels, vitamin D, and calcium, a Pearson correlation coefficient (*r*) was used.

## Results

### Overall Cohort

One hundred sixty patients were included in the analysis with 77 patients having undergone TT, 31 patients having undergone UT, and 52 patients having undergone PT. The average age at the time of surgery was 54.5 years for the thyroidectomy (TT and UT) cohort and 59.3 years for the PT cohort. The majority of patients were female in both the thyroidectomy and PT cohorts (78.7% and 76.2%, respectively). For those in the thyroidectomy cohort, most had malignant pathology with the majority being papillary thyroid carcinoma. All those in the PT cohort had benign pathology (Table 1).

**Table 1:** Patient Demographics

	Thyroidectomy (n = 108)	Parathyroidectomy (n = 52)
Subjects		
Total	72 (66.7%)	‐
Completion	5 (4.6%)	‐
Unilateral	31 (28.7%)	‐
Age, y	54.5 ± 16.1	59.3 ± 14.5
Sex		
Male	23 (21.3%)	10 (23.8%)
Female	85 (78.7%)	42 (76.2%)
*Pathology*		
Malignant	75 (69.4%)	
PTC	68 (63.0%)	‐
FTC	4 (3.7%)	‐
MTC	2 (1.8%)	‐
Anaplastic carcinoma	1 (0.9%)	‐
Benign	33 (30.6%)	52 (100%)

Summary of patient demographics.

Abbreviations: FTC, follicular thyroid carcinoma; MTC, medullary thyroid carcinoma; PTC, papillary thyroid carcinoma.

### PTH Levels

**Figure 1:**
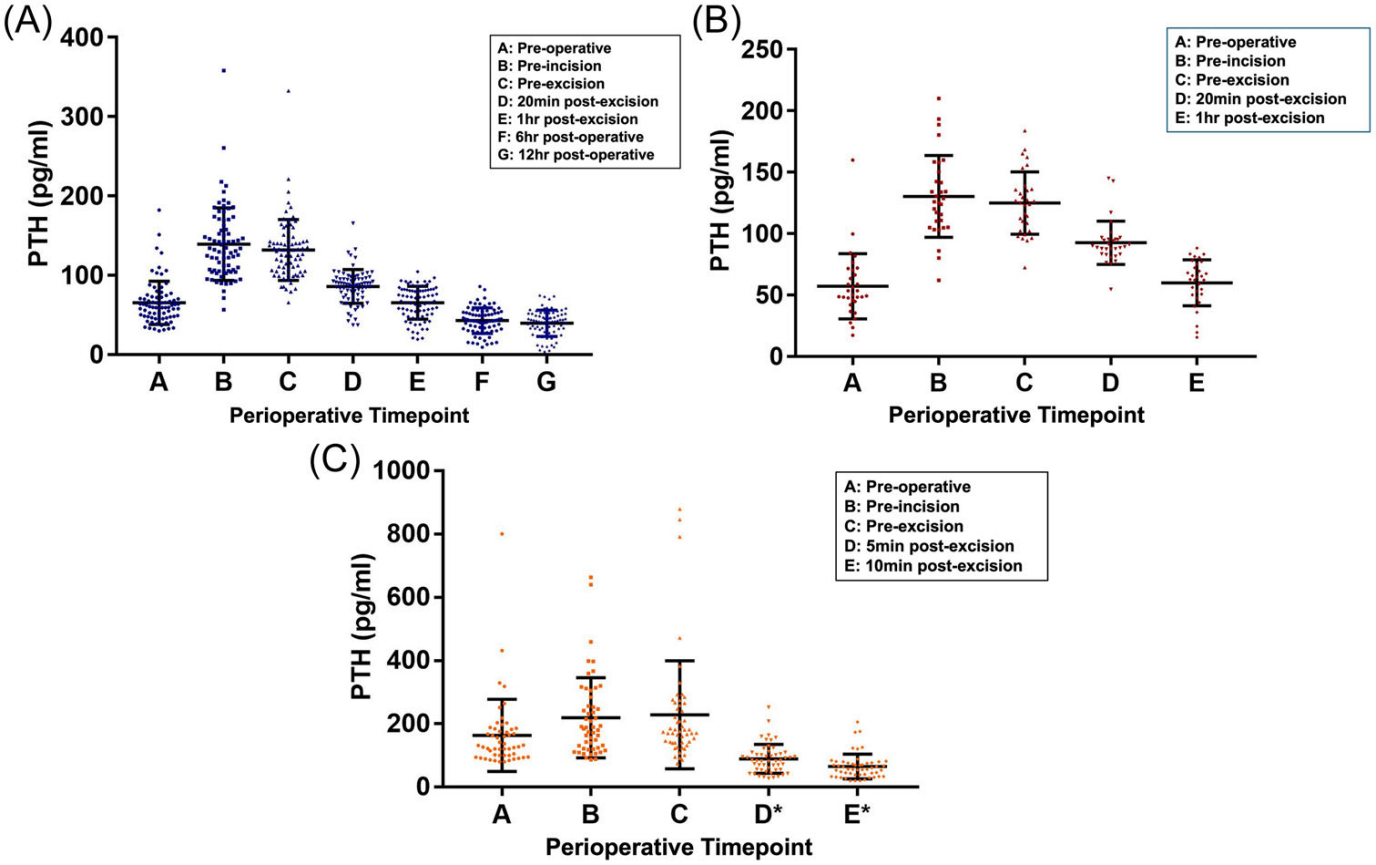
PTH patterns. Dot plots of individual PTH patterns in total thyroidectomy (A), unilateral thyroidectomy (B), and parathyroidectomy (C). A: preoperative, B: preincision, C: pre‐excision, D: 20 minutes postexcision, D*: 5 minutes postexcision (parathyroidectomy only), E: 1 hour postexcision, E*: 10 minutes postexcision (parathyroidectomy only), F: 6 hours postoperative, G: 12 hours postoperative. Note that *x‐*axis (timepoints) is not to scale. Error bars represent standard deviation. PTH, parathyroid hormone.

All patients showed a statistically significant increase in PTH from preoperative to preincision time points in both the TT and UT (*P* < .0001) group, as well as the PT group (*P* = .01). Mean PTH levels were significantly higher following anesthesia induction and endotracheal intubation across all surgical groups. PTH levels remained elevated until excision (TT: 131.8 pg/mL; UT: 124.9 pg/mL; PT: 228.7 pg/mL). Following UT, mean PTH declined to preoperative levels by 1 hour postexcision. There was no significant difference in PTH between TT and UT groups at the preoperative, preincision, pre‐excision, and 20 minutes and 1 hour postexcision time points (*P* value range:.121‐.352). Following UT, mean PTH declined to preoperative levels by 1 hour postexcision (Supplemental Table S1, available online, and Figure 1). In the PT group, patterns of decrease from preoperative to preincision and either subsequent increase or decrease from preincision to pre‐excision occurred in 17% that was not seen in the thyroidectomy groups.

**Figure 2:**
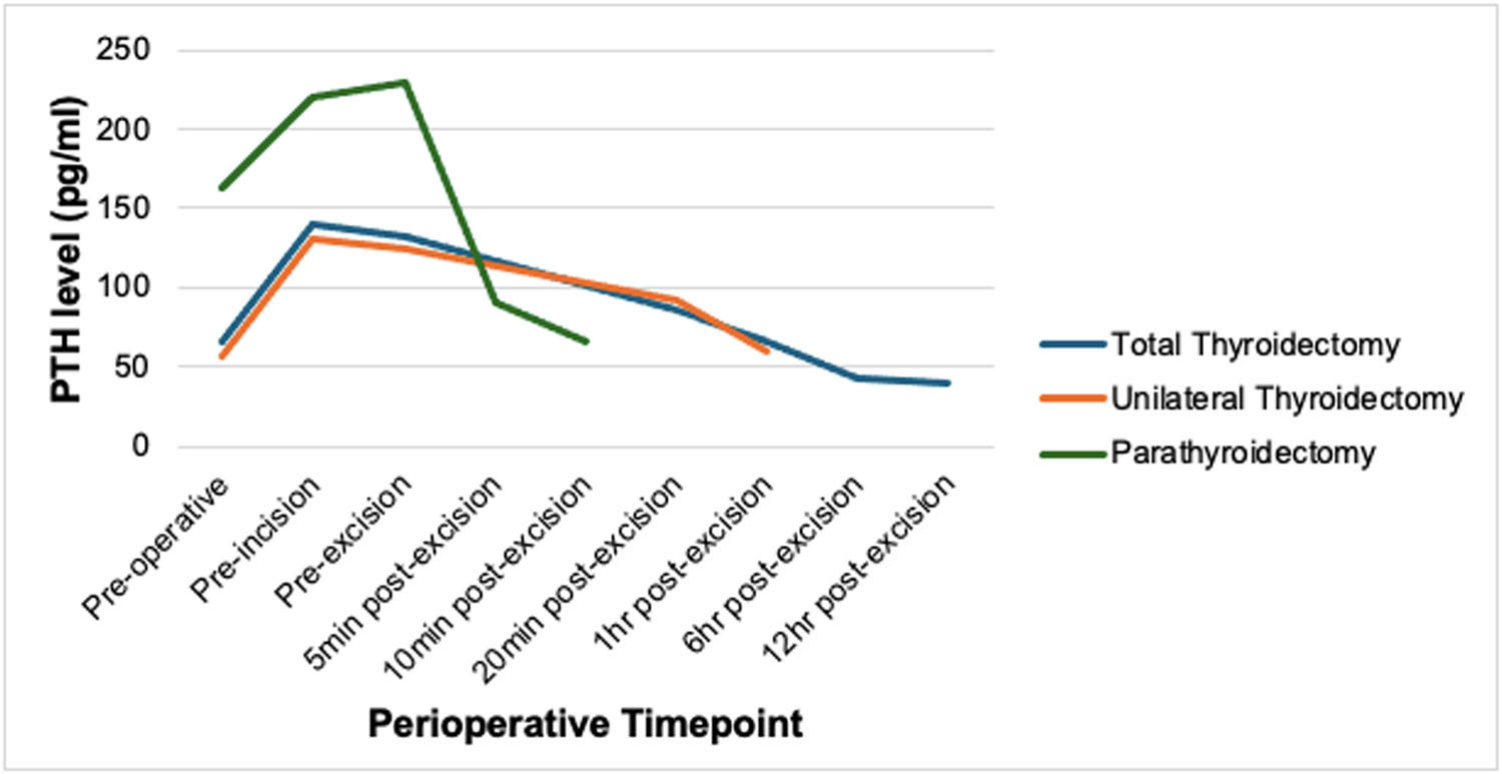
Perioperative PTH changes across surgical groups. The figure demonstrates perioperative PTH changes for the 3 studied surgical groups: total thyroidectomy, unilateral thyroidectomy, and parathyroidectomy. Total thyroidectomy patient PTH levels were measured until 12 hours postexcision. Unilateral thyroidectomy patient PTH levels were measured until 1 hour postexcision. Parathyroidectomy patient PTH levels were measured until 10 minutes postexcision. PTH, parathyroid hormone.

Supplemental Table S1, available online, and Figure 2 present the mean PTH values for each perioperative time point. In both TT and UT, as well as PT, all patients showed a significant increase in PTH levels from preoperative to preincision time points (*P* < .0001 for TT and UT; *P* = .01 for PT). The mean PTH values for TT increased from 65.1 + 27.4 to 139.2 + 45.8 pg/mL (113.8% increase), for UT from 57.1 + 26.5 to 130.4 + 33.3 pg/mL (128.4% increase), and for PT from 163.7 + 114.1 to 219.6 + 126.7 pg/mL (34.1% increase). There was no significant difference in PTH levels between the TT and UT groups at any of the measured time points, including preoperative, preincision, pre‐excision, and 20 minutes and 1 hour postexcision (*P* value range: .121‐.352). The absolute and percentage PTH elevations following anesthesia induction were similar between the 2 groups (*P* = .904 and *P* = .067, respectively).

**Figure 3:**
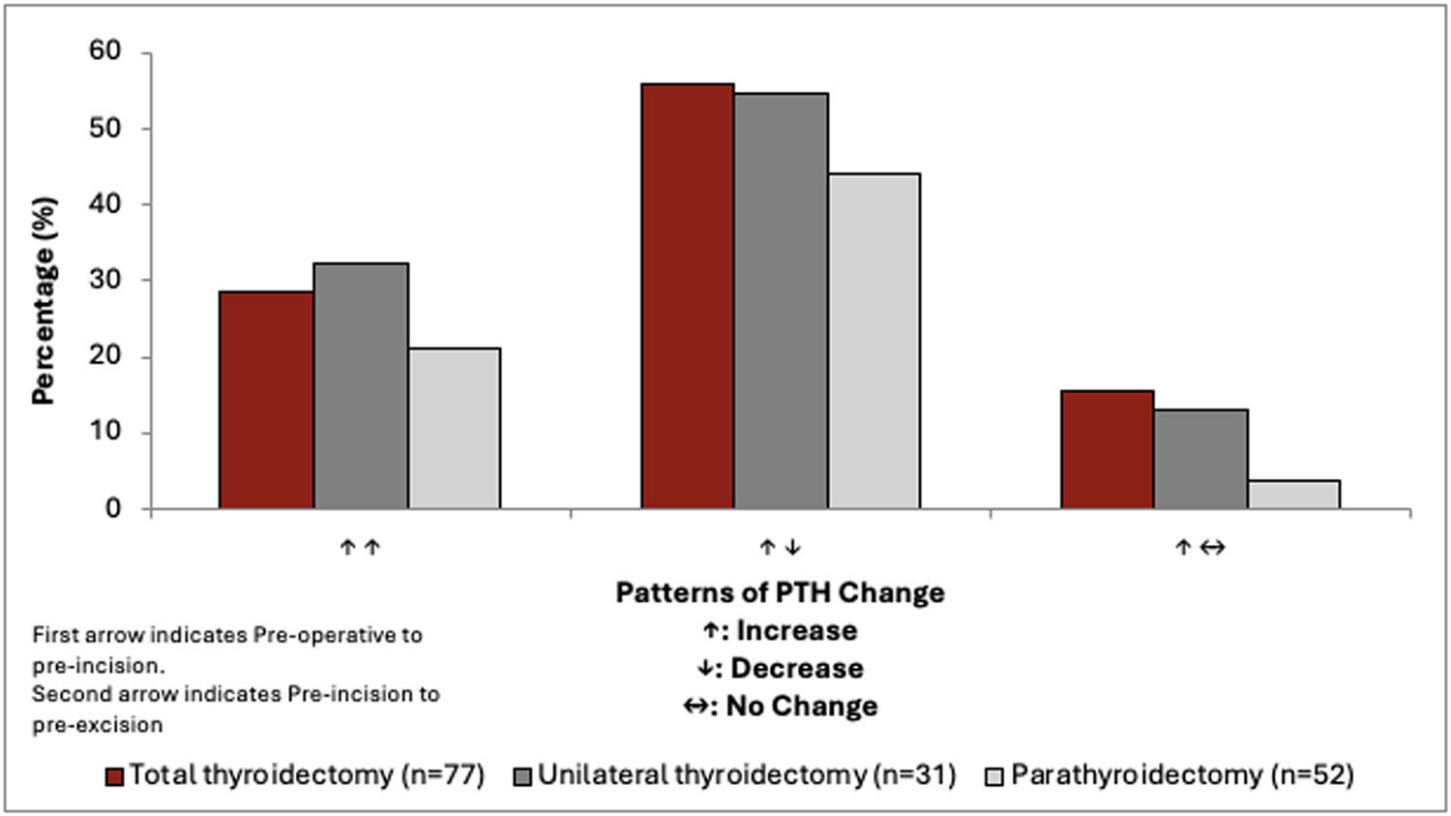
Summarized patterns of PTH change. The figure demonstrates the summarized PTH changes for the timeframes of preoperative to preincision and preincision to pre‐excision using arrows with “↑” being increase, “↓” being decrease, and “↔” being no change. PTH, parathyroid hormone.

Supplemental Table S2, available online, and Figure 3 summarizes the PTH patterns observed at 3 different time points: preoperative, preincision, and pre‐excision. In the TT and UT groups, there was a consistent increase in PTH levels from preoperative to preincision time point. However, the changes in PTH levels from preincision to pre‐excision were less predictable. In the case of PT, there was more variability in PTH patterns from preoperative to preincision time point. Only 69.2% of patients (n = 36) showed an increase in PTH.

In both TT and UT groups, there was no significant difference in mean serum calcium between preoperative and preincision time points (9.5 ± 0.47 vs 9.4 ± 0.34 mg/dL; *P* = .437 for total and 9.5 ± 0.46 vs 9.3 ± 0.31 mg/dL; *P* = .066 for unilateral). Preoperative calcium (9.48 ± 0.47 vs 9.53 ± 0.46 mg/dL; *P* = .554) and 25‐OH vitamin D (30.9 ± 10.2 vs 32.6 ± 10.5 ng/mL; *P* = .415) levels did not differ significantly between the TT and UT groups. Additionally, there were no significant correlations observed between preoperative calcium and 25‐OH vitamin D levels and any of the perioperative PTH levels (Supplemental Table S3, available online). Among the different perioperative time points, only preoperative PTH showed a moderate correlation with preincision and pre‐excision PTH in both TT (*r* = .714; *r* = .647) and UT (*r* = .552; *r* = .508).

Figures 1 and 2 and Supplemental Table S1, available online, depict the PTH trends of thyroidectomy patients as a whole and individually. PTH remained elevated at the pre‐excision time point following skin incision, with mean PTH levels of 131.8 + 38.2 pg/mL in TT and 124.9 + 25.5 pg/mL in UT. Supplemental Table S4, available online, shows that 29 TT and 12 UT patients experienced further increases in PTH compared to preincision values. Following thyroid excision, the majority of patients (96.3%) showed a decrease in PTH, with mean PTH levels returning to preoperative values (65.1 + 27.4 pg/mL in TT; 57.1 + 26.5 pg/mL in UT) by 1 hour postexcision (65.2 + 20.8 pg/mL in TT; 59.9 + 18.8 pg/mL in UT; Supplemental Table S1, available online). At 20 minutes to 1 hour postexcision, all but 1 patient in the TT group had a drop in PTH. After TT, PTH levels remained stable at 12 hours postoperatively, with an average hourly PTH change rate of −0.539 ± 2.52 pg/mL/h from 6 to 12 hours compared to −3.08 + 2.37 pg/mL/h from 1 to 6 hours postthyroidectomy.

We then investigated the relationship between eGFR and PTH levels as well as rates of PTH change in thyroidectomy patients. Out of the TT and UT groups, 59 and 25 patients, respectively, had available eGFR data for analysis. The mean preoperative eGFR did not significantly differ between the 2 groups (90.9 ± 16.1 mL/min/1.73 m^2^ and 96.3 ± 10.5 mL/min/1.73 m^2^, respectively; *P* = .128). PTH levels were analyzed based on different stages of CKD, but overall, there was no correlation between eGFR and PTH levels at any perioperative time point (Supplemental Table S5, available online). Additionally, there was no significant difference in PTH levels among various stages of CKD at each perioperative time point. The rate of PTH change was compared based on different stages of CKD at each time point (Supplemental Table S6, available online). The study found that there was no significant relationship between eGFR and the rate of PTH change in both TT and UT groups, and the degree of CKD did not significantly affect the rate of PTH change at each interval.

## Discussion

Perioperative PTH levels have been used to determine the effectiveness of PT in patients with hyperparathyroidism and to predict postoperative hypocalcemia in patients after total or near‐TT.^19^ In this study, we studied the effects of anesthetic and surgical stress on those with normal parathyroid function undergoing UTS/TTS and PTs. All patients undergoing thyroidectomy displayed increases in PTH following anesthesia induction and intubation. This is consistent with PTH elevation secondary to the activation of stress response via alpha‐adrenergic stimulation shown in previous studies.^2^, ^12^, ^20^ The changes in PTH following anesthesia induction, however, were not correlated with serum calcium level changes. In the study by Hong et al, general endotracheal anesthesia was shown to cause the highest increase in PTH compared to laryngeal mask airway and monitored anesthesia care, although in patients with hyperparathyroidism undergoing PT.^2^

This investigation of thyroidectomy cases with normal parathyroid function reveals that PTH consistently increases in adults after initial anesthesia induction. In contrast, those with hyperparathyroidism demonstrated variable PTH changes to anesthesia and surgery. Mizrachi et al looked at PTH levels after PT in patients with hyperparathyroidism.^21^ In their cohort, they found variable results with only 25% of patients having elevated PTH levels. This is consistent with our result that patients with hyperparathyroidism had varying changes to anesthesia and surgery. Cinamon et al found that GA caused a significant PTH increase in patients with hyperparathyroidism and only a mild increase in healthy adults, contrary to our findings.^16^ However, they looked at endotracheal intubation in addition to GA in patients undergoing nonhead and neck surgeries. The stress of passing an endotracheal tube can cause an increase in catecholamines and PTH rather than GA itself.^22^ Additionally, our study looked specifically at healthy adults undergoing thyroidectomies and PTs.

Operating in the area of the neck where PTH is produced can result in a more pronounced effect than elective surgeries elsewhere. Intraoperative manipulation of parathyroid adenomas, such as through mechanical stimulation (squeezing or manual rubbing), has been shown to lead to increased PTH secretion. A study by Riss et al documented that manual manipulation of parathyroid adenomas resulted in a significant rise in PTH levels in 4 out of 6 patients, with 1 patient's PTH level increasing from 343 to 1747 pg/mL.^23^ Additionally, unintended manipulation of parathyroid adenomas during surgery for primary hyperparathyroidism can frequently cause “PTH spikes,” complicating the interpretation of intraoperative PTH monitoring.^24^ This phenomenon underscores the importance of careful handling of the parathyroid glands during surgical procedures to avoid misleading intraoperative PTH elevations.

Furthermore, elevated perioperative PTH levels in cases with recent normal outpatient levels do not necessarily imply hyperparathyroidism in those undergoing thyroidectomy. PTH levels earlier than 1 hour postexcision (when mean PTH first approximates preoperative levels) may not be indicative or predictive of parathyroid function after TT or PT. Following thyroidectomy, PTH followed a downward trend in general, except in 4 patients.

PTH levels are known to be influenced by calcium levels, undergo clearance with a less than 4‐minute half‐life, with increases due to manipulation of intact parathyroid glands, and decreases due to parathyroid gland removal or injury, and parathyroid gland blood supply compromise.^23^, ^24^ This study shows that the additional factor of PTH increase related to GA occurs in both PT with abnormal parathyroid function and thyroidectomy patients with normal parathyroid function and should be considered when measuring PTH levels perioperatively. This phenomenon helps explain cases of prolonged decline to normal levels after satisfactory PT. In these cases of failure to meet Miami criteria at 10 minutes resulting from anesthesia‐related PTH elevations, decay of PTH may not occur as quickly as expected, so minimizing anesthesia and repeating PTH rather than further exploration may be prudent. Elevated PTH levels after TT particularly when still under GA such as during closure or if additional procedures such as lateral neck dissections are performed following thyroidectomy may raise suspicion of hyperparathyroidism and result in unwarranted parathyroid exploration. Preoperative parathyroid function assessment and waiting until the postanesthetic period to examine PTH levels would allay this concern. No patients with elevated post‐TT PTH levels exhibited hypocalcemic sequela or subsequent low or undetectable PTH levels.

One limitation of our study is that the cohorts consisted of a majority of female patients. Estrogen and progesterone are known to stimulate PTH secretion, indicating that women typically have higher baseline serum PTH levels than men.^25^ Future studies should explore potential gender differences in baseline PTH levels and investigate how these differences may influence the perioperative changes in PTH levels following thyroidectomy and PT. In addition to preoperative parathyroid function, many studies have found other factors that could affect IOPTH, such as kidney function and seasonal changes.^18^, ^26^, ^27^ The analysis of PTH patterns in relation to eGFR is limited by a small sample size in some subgroups (eg, only 4 patients with eGFR <60) thus limiting the conclusions that can be drawn. Future research should continue to explore additional factors that contribute to IOPTH level changes and risk factors for postthyroidectomy hypocalcemia.

## Conclusion

PTH increases in response to anesthetic and surgical stress in adults with normal preoperative parathyroid function undergoing UT and TT. Elevated PTH levels during and after thyroidectomy are common and not always an indication of undiagnosed hyperparathyroidism if there was no preoperative indication of such.

## Author Contributions

**Emily S. Sagalow**, was involved in analysis, drafting, manuscript review, and revision; **Yuna Kim**, was involved in design, analysis, drafting, manuscript review, and revision; **Shirley Wong**, was involved in drafting, manuscript review, and revision; **Kavita Batra**, was involved in manuscript review and revision; **Annabel Barber**, was involved in manuscript review and revision; **Robert C. Wang**, was involved in conception, design, data acquisition, analysis, drafting, manuscript review, and revision. All authors approved the final version of the manuscript to be published.

## Disclosures

### Competing interests

The authors report no proprietary or commercial interest in any product mentioned or concept discussed in this article. This research did not receive any specific grant from funding agencies in the public, commercial, or not‐for‐profit sectors.

### Funding source

None.

## Supporting information

Supporting information.
