# Supplementary material for: Parathyroid Hormone Fluctuations During Thyroid and Parathyroid Surgery
Source: OTO Open. 2025 Jan 17;9(1):e70068. doi: 10.1002/oto2.70068 (PMC11739895; doi:10.1002/oto2.70068)
Supplement: Supplementary file 1 — Supporting information. [file OTO2-9-e70068-s001.docx]

**Supplemental Table 1: Perioperative PTH Changes Across Surgical Groups**

| **PTH (pg/ml)** | **TT (n=77)** | **UT (n=31)** | **PT (n=52)** |
| --- | --- | --- | --- |
| **Pre-operative** | 65.1 ± 27.4 | 57.1 ± 26.5 | 163.7 ± 114.1 |
| **Pre-incision** | 139.2 ± 45.8  **(p<0.0001)** | 130.4 ± 33.3  **(p<0.0001)** | 219.6 ± 126.7  **(p=0.01)** |
| *Mean absolute increase (pg/ml)* | *74.1 ± 32.5* | *73.3 ± 29.0* | *55.9 ± 83.9* |
| *Mean percentage increase (%)* | *127.7 ± 64.7* | *155.8 ± 86.1* | *43.3 ± 55.2* |
| **Pre-excision** | 131.8 ± 38.2 | 124.9 ± 25.5 | 228.7 ± 171.1 |
| **5min post-excision** | ─ | ─ | 89.6 ± 45.9 |
| **10min post-excision** | ─ | ─ | 65.4 ± 39.1 |
| **20min post-excision** | 85.8 ± 21.4 | 92.5 ± 17.5 | ─ |
| **1hr post-excision** | 65.2 ± 20.8 | 59.9 ± 18.8 | ─ |
| **6hr post-operative** | 42.7 ± 16.2 | ─ | ─ |
| **12hr post-operative** | 39.4 ± 16.5 | ─ | ─ |

Summary of mean PTH levels at various perioperative time points across all surgical groups (TT: Total thyroidectomy, UT: Unilateral thyroidectomy, PT: Parathyroidectomy).

**Supplemental Table 2: Patterns of PTH Change in Surgical Groups**

| **Patterns of PTH Change** | | **Surgery Group** | | |
| --- | --- | --- | --- | --- |
| **Pre-operative to pre-incision PTH** | **Pre-incision to pre-excision PTH** | **TT (n=77)** | **UT (n=31)** | **PT (n=52)** |
| ↑ | ↑ | 22 (28.6%) | 10 (32.3%) | 11 (21.2%) |
| ↑ | ↓ | 43 (55.8%) | 17 (54.8%) | 23 (44.2%) |
| ↓ | ↑ | 0 | 0 | 6 (11.5%) |
| ↓ | ↓ | 0 | 0 | 3 (5.8%) |
| ↑ | ↔ | 12 (15.6%) | 4 (12.9%) | 2 (3.8%) |
| ↔ | ↑ | 0 | 0 | 3 (5.8%) |
| ↓ | ↔ | 0 | 0 | 0 |
| ↔ | ↓ | 0 | 0 | 3 (5.8%) |
| ↔ | ↔ | 0 | 0 | 1 (1.9%) |

Patterns of PTH change in all surgical groups (The margin of error for the PTH assay analyzer was ≤ 4%, and any absolute change in PTH of 4% or less was considered no change “↔”).

**Supplemental Table 3: Perioperative PTH Correlation with Pre-operative PTH, Calcium, and Vitamin D**

|  | **Pearson correlation coefficient (r)** | | | | | | |
| --- | --- | --- | --- | --- | --- | --- | --- |
| **Group** | **Pre-operative PTH** | **Pre-incision PTH** | **Pre-excision PTH** | **20min post-excision PTH** | **1hr post-excision PTH** | **6hr**  **post-operative PTH** | **12hr**  **post-operative PTH** |
| **Total** |  | | | | | | |
| Pre-operative PTH | 1.00 | 0.714 | 0.647 | 0.394 | 0.225 | 0.370 | 0.280 |
| Ca | −0.050 | −0.042 | 0.045 | 0.040 | −0.003 | 0.076 | −0.002 |
| 25-OH vitamin D | 0.080 | 0.175 | 0.102 | 0.125 | 0.212 | 0.135 | 0.078 |
| **Unilateral** |  | | | | | | |
| Pre-operative PTH | 1.00 | 0.552 | 0.508 | 0.201 | 0.238 | ─ | ─ |
| Ca | 0.091 | 0.132 | 0.139 | −0.009 | −0.026 | ─ | ─ |
| 25-OH vitamin D | −0.158 | −0.004 | −0.079 | 0.281 | 0.107 | ─ | ─ |

Correlation between pre-operative PTH, calcium and 25-OH vitamin D vs. various perioperative PTH levels in thyroidectomy groups.

**Supplemental Table 4: Perioperative Summary of PTH Change**

|  | **Mean PTH change per hour (pg/ml/hr)** | | | | | |
| --- | --- | --- | --- | --- | --- | --- |
| **Group** | **Pre-operative to pre-incision** | **Pre-incision to pre-excision** | **Pre-excision to 20min post-excision** | **20min to 1hr post-excision** | **1hr post-excision to 6hr post-operative** | **6hr to 12hr post-operative** |
| **Total (n=77)** |  | | | | | |
| (range) | 52.3 ± 26.1  (10.0 to 116.1) | −13.2 ± 34.2  (−123.1 to 55.3) | −28.3 ± 22.4  (−142.5 to 4.11) | −30.7 ± 23.3  (−107.6 to 24.6) | −3.08 ± 2.37  (−10.3 to 3.93) | −0.539 ± 2.52  (−9.33 to 5.17) |
| **Slope** | **n** | | | | | |
| >0 | 77 | 29 | 3 | 1 | 5 | 35 |
| <0 | 0 | 48 | 74 | 76 | 72 | 42 |
| **Unilateral (n=31)** |  | | | | | |
| (range) | 54.0 ± 26.0  (21.0 to 114.1) | −14.0 ± 38.3  (−115.9 to 54.1) | −30.6 ± 21.5  (−102.1 to 9.08) | −48.6 ± 27.0  (−105.7 to −4.62) | ─ | ─ |
| **Slope** | **n** | | | | | |
| >0 | 31 | 12 | 1 | 0 | ─ | ─ |
| <0 | 0 | 19 | 30 | 31 | ─ | ─ |

Summary of rates of PTH change between various consecutive perioperative intervals.

**Supplemental Table 5: eGFR-Stratified Analysis of Rates of PTH Change Pre-operatively**

|  | **Mean PTH (pg/ml)** | | | | | | |
| --- | --- | --- | --- | --- | --- | --- | --- |
| **eGFR (ml/min/1.73m^2^)** | **Pre-operative** | **Pre-incision** | **Pre-excision** | **20min post-excision** | **1hr**  **post-excision** | **6hr**  **post-operative** | **12hr post-operative** |
| **Total** |  | | | | | | |
| All eGFR (n=59) | 66.8 ± 30.5 | 142.0 ± 50.3 | 134.1 ± 42.1 | 82.4 ± 23.0 | 59.8 ± 20.6 | 39.8 ± 16.8 | 37.1 ± 17.7 |
| *≥90 (n=37)* | *64.2 ± 31.6* | *138.6 ± 57.3* | *130.4 ± 47.1* | *83.6 ± 26.6* | *62.1 ± 19.3* | *40.7 ± 15.6* | *35.8 ± 17.9* |
| *60-89 (n=18)* | *69.8 ± 30.5* | *150.1 ± 35.3* | *140.6 ± 29.9* | *81.3 ± 15.6* | *56.9 ± 24.9* | *39.6 ± 19.9* | *41.2 ± 18.4* |
| *<60 (n=4)* | *76.2 ± 21.6* | *137.6 ± 43.7* | *139.2 ± 45.2* | *76.0 ± 18.5* | *52.5 ± 8.85* | *33.0 ± 15.9* | *30.9 ± 9.36* |
| **(F ratio)** | 0.400 | 0.375 | 0.378 | 0.396 | 0.655 | 0.370 | 0.832 |
| **(P value)** | 0.672 | 0.689 | 0.687 | 0.675 | 0.524 | 0.693 | 0.441 |
|  | ***r*** | | | | | | |
| eGFR | −0.103 | −0.045 | −0.108 | 0.080 | 0.163 | 0.189 | 0.073 |
| **Unilateral** |  | | | | | | |
| All eGFR (n=25) | 56.0 ± 28.8 | 130.2 ± 36.6 | 124.9 ± 27.7 | 92.5 ± 19.1 | 58.1 ± 20.0 | ─ | ─ |
| *≥90 (n=21)* | *59.0 ± 29.5* | *132.2 ± 39.3* | *126.2 ± 29.6* | *92.8 ± 20.8* | *59.0 ± 20.5* | *─* | *─* |
| *60-89 (n=4)* | *40.2 ± 21.4* | *119.8 ± 15.5* | *118.1 ± 14.6* | *91.1 ± 5.95* | *53.2 ± 18.4* | *─* | *─* |
| **(T value)** | 1.207 | 0.614 | 0.527 | 0.122 | 0.529 | ─ | ─ |
| **(P value)** | 0.120 | 0.273 | 0.302 | 0.452 | 0.301 | ─ | ─ |
|  | ***r*** | | | | | | |
| eGFR | 0.206 | 0.080 | 0.030 | −0.120 | −0.042 | ─ | ─ |

Stratified analysis of perioperative PTH levels based on pre-operative eGFR in total and unilateral thyroidectomy (r = Pearson correlation coefficient).

**Supplemental Table 6: eGFR-Stratified Analysis of Rates of PTH Change Perioperatively**

|  | **Mean PTH change per hour (pg/ml/hr)** | | | | | |
| --- | --- | --- | --- | --- | --- | --- |
| **eGFR (ml/min/1.73m^2^)** | **Pre-operative to pre-incision** | **Pre-incision to pre-excision** | **Pre-excision to 20min post-excision** | **20min to 1hr post-excision** | **1hr post-excision to 6hr post-operative** | **6hr to 12hr post-operative** |
| **Total (n=59)** |  | | | | | |
| All eGFR  (range) | 53.0 ± 26.9 (10.0 to 116.1) | −14.4 ± 37.7  (−123.1 to 55.3) | −31.7 ± 23.9  (−142.5 to 4.11) | −33.3 ± 24.8  (−107.6 to 24.6) | −2.81 ± 2.55 (−10.3 to 3.93) | −0.484 ± 2.82  (−9.33 to 5.17) |
| *≥90* | *51.0 ± 23.8* | −*13.7 ± 36.8* | −*28.6 ± 25.6* | −*32.0 ± 23.1* | −*2.91 ± 1.98* | −*0.817 ± 2.86* |
| *60-89* | *60.8 ± 30.4* | −*19.4 ± 40.2* | −*36.6 ± 20.6* | −*36.5 ± 28.8* | −*2.57 ± 3.72* | *0.269 ± 2.76* |
| *<60* | *43.1 ± 37.9* | *5.74 ± 41.1* | −*39.7 ± 23.3* | −*35.0 ± 28.0* | −*2.53 ± 0.978* | −*0.363 ± 3.12* |
| **Slope** | **n** | | | | | |
| >0 | 59 | 24 | 3 | 1 | 5 | 31 |
| <0 | 0 | 35 | 56 | 58 | 54 | 28 |
| **(F ratio)** | 1.13 | 0.715 | 1.07 | 0.075 | 0.123 | 0.883 |
| **(P value)** | 0.330 | 0.493 | 0.350 | 0.928 | 0.885 | 0.419 |
|  | **r** | | | | | |
| eGFR | -0.025 | -0.096 | 0.147 | 0.120 | 0.030 | -0.105 |
| **Unilateral (n=25)** |  | | | | | |
| All eGFR  (range) | 56.6 ± 28.3  (21.0 to 114.1) | −14.2 ± 42.1  (−115.9 to 54.1) | −31.5 ± 23.6  (−102.1 to 9.08) | −51.4 ± 28.6  (−105.7 to −4.62) | ─ | ─ |
| *≥90* | *53.8 ± 29.7* | −*15.5 ± 44.5* | −*32.5 ± 25.2* | −*50.4 ± 28.9* | *─* | *─* |
| *60-89* | *71.4 ± 12.5* | −*7.58 ± 30.8* | −*26.5 ± 13.4* | −*56.6 ± 30.8* | *─* | *─* |
| **Slope** | **n** | | | | | |
| >0 | 25 | 11 | 1 | 0 | ─ | ─ |
| <0 | 0 | 14 | 24 | 25 | ─ | ─ |
| **(T value)** | −1.146 | −0.337 | −0.492 | 0.416 | ─ | ─ |
| **(P value)** | 0.133 | 0.370 | 0.314 | 0.341 | ─ | ─ |
|  | **r** | | | | | |
| eGFR | −0.190 | −0.126 | −0.182 | 0.076 | ─ | ─ |

eGFR-stratified analysis of rates of PTH change between various consecutive perioperative intervals in total and unilateral thyroidectomy (r = Pearson correlation coefficient).
